# Identification, characterization and gene expression analyses of important flowering genes related to photoperiodic pathway in bamboo

**DOI:** 10.1186/s12864-018-4571-7

**Published:** 2018-03-10

**Authors:** Smritikana Dutta, Prasun Biswas, Sukanya Chakraborty, Devrani Mitra, Amita Pal, Malay Das

**Affiliations:** 1 Department of Life Sciences, Presidency University, Kolkata, India; 2 Division of Plant Biology, Bose Institute, Kolkata, India

**Keywords:** Bamboo, Flowering genes, Circadian clock, Photoperiodism, Gene expression

## Abstract

**Background:**

Bamboo is an important member of the family Poaceae and has many inflorescence and flowering features rarely observed in other plant groups. It retains an unusual form of perennialism by having a long vegetative phase that can extend up to 120 years, followed by flowering and death of the plants. In contrast to a large number of studies conducted on the annual, reference plants *Arabidopsis thaliana* and rice, molecular studies to characterize flowering pathways in perennial bamboo are lacking. Since photoperiod plays a crucial role in flower induction in most plants, important genes involved in this pathway have been studied in the field grown *Bambusa tulda*, which flowers after 40-50 years.

**Results:**

We identified several genes from *B. tulda*, including four related to the circadian clock [*LATE ELONGATED HYPOCOTYL* (*LHY*), *TIMING OF CAB EXPRESSION1* (*TOC1*), *ZEITLUPE* (*ZTL*) and *GIGANTEA* (*GI*)], two circadian clock response integrators [*CONSTANS A* (*COA*), *CONSTANS B* (*COB*)] and four floral pathway integrators [*FLOWERING LOCUS T1, 2, 3, 4* (*FT1, 2, 3, 4*)]. These genes were amplified from either gDNA and/or cDNA using degenerate as well as gene specific primers based on homologous sequences obtained from related monocot species. The sequence identity and phylogenetic comparisons revealed their close relationships to homologs identified in the temperate bamboo *Phyllostachys edulis*. While the four *BtFT* homologs were highly similar to each other, *BtCOA* possessed a full-length B-box domain that was truncated in *BtCOB*. Analysis of the spatial expression of these genes in selected flowering and non-flowering tissue stages indicated their possible involvement in flowering. The diurnal expression patterns of the clock genes were comparable to their homologs in rice, except for *BtZTL*. Among multiple *BtCO* and *BtFT* homologs, the diurnal pattern of only *BtCOA* and *BtFT3*, *4* were synchronized in the flower inductive tissue, but not in the non-flowering tissues.

**Conclusion:**

This study elucidates the photoperiodic regulation of bamboo homologs of important flowering genes. The finding also identifies copy number expansion and gene expression divergence of *CO* and *FT* in bamboo. Further studies are required to understand their functional role in bamboo flowering.

**Electronic supplementary material:**

The online version of this article (10.1186/s12864-018-4571-7) contains supplementary material, which is available to authorized users.

## Background

Controlling flowering time is one of the most important adaptations linked to the survival of angiosperms. Annual plants like *A. thaliana* or rice (*Oryza sativa*) undergo a short vegetative phase of a few weeks before the onset of flowering and then die. On the other hand, woody perennials such as *Populus* undergo years of vegetative growth before the onset of flowering and the flowering cycle then repeats for successive years. One extreme example of delayed flowering is bamboo, which has a vegetative phase of up to 120 years, followed by flowering and death of the plants [1]. This is a unique biological phenomenon known as semelparity/monocarpy. The flowering incidence may be restricted to few culms of a population (sporadic flowering) [2] or may happen across populations over a large geographical area (gregarious flowering) [3]. An important consequence of gregarious flowering is enormous seed setting, which results in a rapid increase in rat populations and thereby enormous crop loss in the vicinity that might culminate in famine [4]. The sudden induction of flowering also results in disappearance of large areas of vegetation that creates a major ecological imbalance in the surrounding plant community [5, 6]. Therefore, development of molecular markers for detecting possible induction of flowering will be of great help for proper forest management and ensuring food safety.

Flowering is a natural outcome of plant’s interaction with its surrounding environment. Depending on the nature of the external factors various flowering pathways such as photoperiodic (light as external cue) [7], vernalization (cold) [8], autonomous (endogenous factor/s) [9] and hormonal (GA_3_) [10] pathways have been characterized. Light is one of the most studied external cues and can control diverse physiological processes including flowering [11]. In photoperiodic regulation, the duration of day and night governs the timing of flowering, and plants can be categorized as long-day (LDP), short-day (SDP) or day neutral (DNP) [12]. The regulation of flowering as a consequence of day length is governed by the circadian oscillation of the expression of a group of genes known as circadian clock regulated genes [7]. The oscillation of the circadian clock regulated genes in response to light is synchronized by another set of genes called circadian clock genes [13]. In rice *TIMING OF CAB EXPRESSION1* (*TOC1*), *LATE ELONGATED HYPOCOTYL* (*LHY*), *ZEITLUPE* (*ZTL*) and *GIGANTEA* (*GI*) are the major circadian clock genes that have been characterized so far [7–13]. *CONSTANS* (*CO*) is the gene that integrates the clock responses and subsequently passes the signal to the floral pathway integrator gene *FLOWERING LOCUS T* (*FT*) to induce flowering [14, 15]. *CO* is a B-box family gene, having a conserved CCT domain, while *FT* is a member of the phosphatidyl ethanolamine binding protein (PEBP) family.

All these studies have been conducted on the reference dicotyledonous plant *A. thaliana* and monocotyledonous plant rice [11, 16]. These plants have been preferred since they can be easily grown in the laboratory, their growth stages are defined, life cycles are short, germplasms easily accessible, genomes have been sequenced, and several gene mutants are available. However, it is an open question how much of the information generated from these reference plants can be translated to the non-reference plants such as bamboo that possesses striking differences in terms of growth and development. In spite of severe practical limitations such as infrequent tissue availability, low RNA yield, insufficient knowledge regarding floral histology, presence of multiple closely related paralogous flowering genes, woody bamboos offer a very interesting system to study the evolution and functional diversities of flowering genes [17].

Bamboo is a large plant group representing 1441 species within 116 genera and can grow in diverse tropical and temperate habitats [18]. *Phyllostachys heterocycla*, a temperate plant, is the only bamboo that has had its genome sequenced to date [19]. In addition to this small amount of genomic information, de novo transcriptome sequencing has been carried out to generate floral specific expressed sequence tags (ESTs) from different bamboo species such as, *Bambusa oldhamii*, *Dendrocalamus latiflorus*, *P. heterocycla*, *P. edulis*, *P. aurea*, *B. edulis*, *Guadua inermis*, *Otatea acuminata* and *Lithachne pauciflora* [20–27], and limited bamboo flowering genes were functionally characterized using transgenic approaches [28–32]. The transcriptome studies have identified millions of short ESTs of 75-250 bp long. However, in absence of the full-length gene sequences and their detailed functional characterization, understanding of their roles in flowering pathways remains incomplete.

The main objective of this study is to identify, characterize sequences, and analyse expression of important circadian clock and photoperiodic genes in bamboo. Taken together this study presents a comprehensive analysis of a set of flowering pathway genes in *B. tulda*, which flowers after 40-50 years [2].

## Results

### Study of *B. tulda* inflorescence to select appropriate flowering and associated leaf tissues

**Fig. 1:**
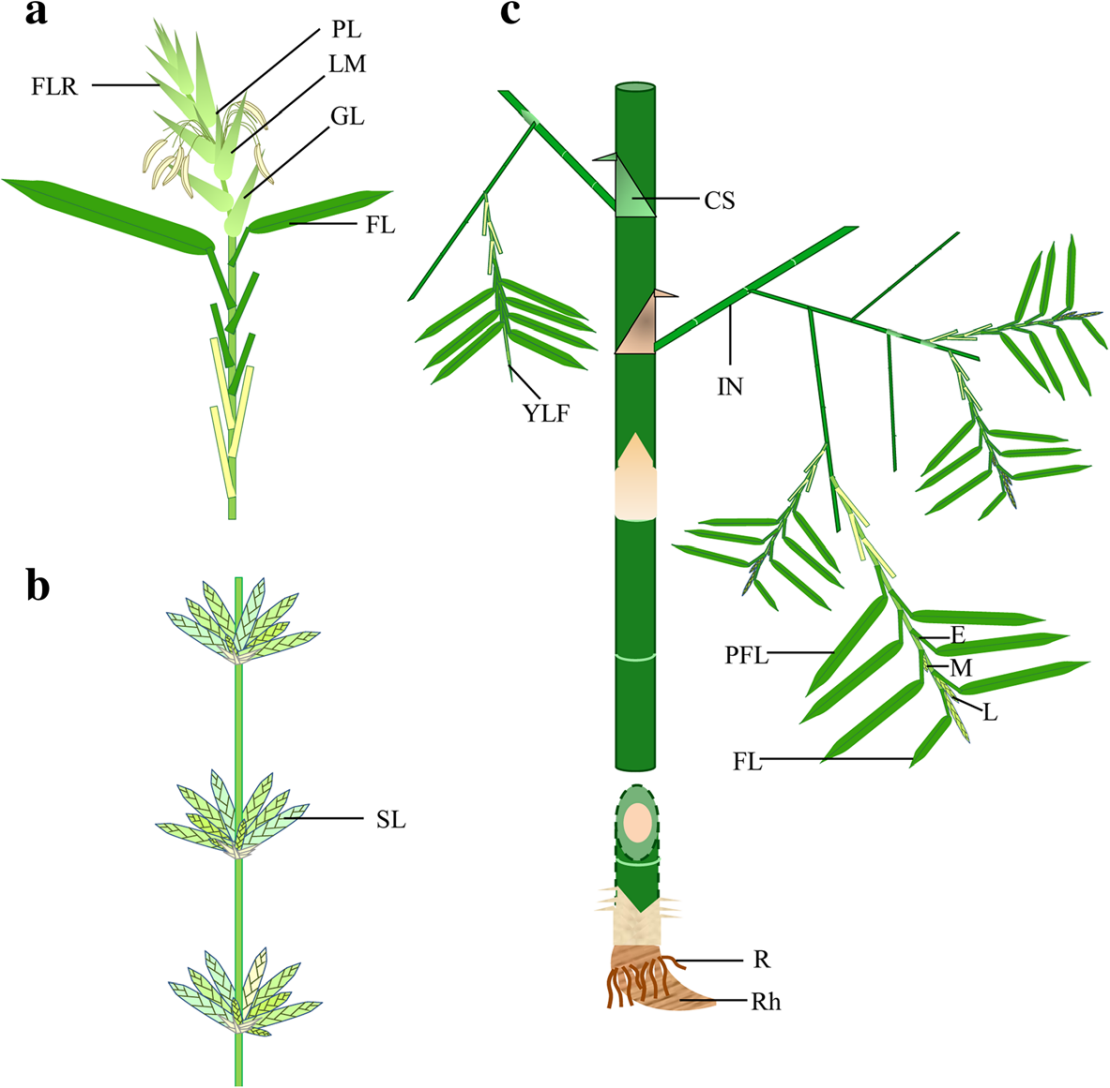
Study of *B. tulda* inflorescence and selection of appropriate flowering and vegetative tissue stages. **a** Morphology of a spikelet in *B. tulda* depicting multiple florets arranged on a single rachis. **b** Morphology of a pseudospikelet in *B. tulda* depicting multiple spikelets arranged in whorls on a rachis. Within each spikelet the florets are arranged on a single rachilla. **c** Different flowering and non-flowering tissue stages selected for studying expression pattern of important flowering genes in *B. tulda*. The Figures were prepared in Microsoft Power point 2016 based on the observations of the plant parts in their natural habitat. The abbreviations used: CS- culm sheath, IN- inter node, YLF- young leaf from flowering culm, YLN- young leaf from non-flowering culm, PFL- possible flag leaf, FL- flag leaf, E- early staged inflorescence bud, M- middle staged inflorescence bud, L- late staged inflorescence bud, R- root, RH- rhizome, PSL- pseudo spikelet, GL- glume, LM- lemma, PL- palea, FLR- floret

Photoperiodic genes are usually regulated by light and hence are expressed primarily in leaves or shoot apex regions [7]. Flowering *B. tulda* plants were observed closely to identify diverse types of leaves that could be studied to understand the photoperiodic regulation of the targeted genes. Like other Poaceae members, the bamboo inflorescence is primarily composed of spikelets, although pseudospikelets are often observed (Fig. 1). Although the bamboo inflorescence is broadly similar to the other two well characterized monocots, rice and maize, yet there exist differences with respect to the position and organization of the inflorescences. For example, in rice the typical flag leaf (FL) is located just beneath the single, terminal inflorescence, while in bamboo a single branch may bear multiple inflorescences, each of which is subtended by an individual FL (Fig. 1). At the advanced flowering stage, several inflorescences develop in a basipetal manner. Young bamboo inflorescences remain covered by the leaf sheath of the FL. As the young inflorescences remain invisible in the early developmental stage, these leaves were defined as possible flag leaves (PFL). Other than FL and PFL, young leaves located in the non-flowering branch of the flowering culm (YLF) may play a role in floral induction and therefore were included in the present study. In addition, a few more vegetative tissues were included such as culm sheath (CS), internodal region (IN), young leaf from non-flowering culm (YLN), root (R), rhizome (RH) and also reproductive tissues such as early staged inflorescence bud (E), middle staged inflorescence bud (M), late staged inflorescence bud (L, Fig. 1).

### Molecular identification and sequence characterization of circadian clock genes

**Fig. 2:**
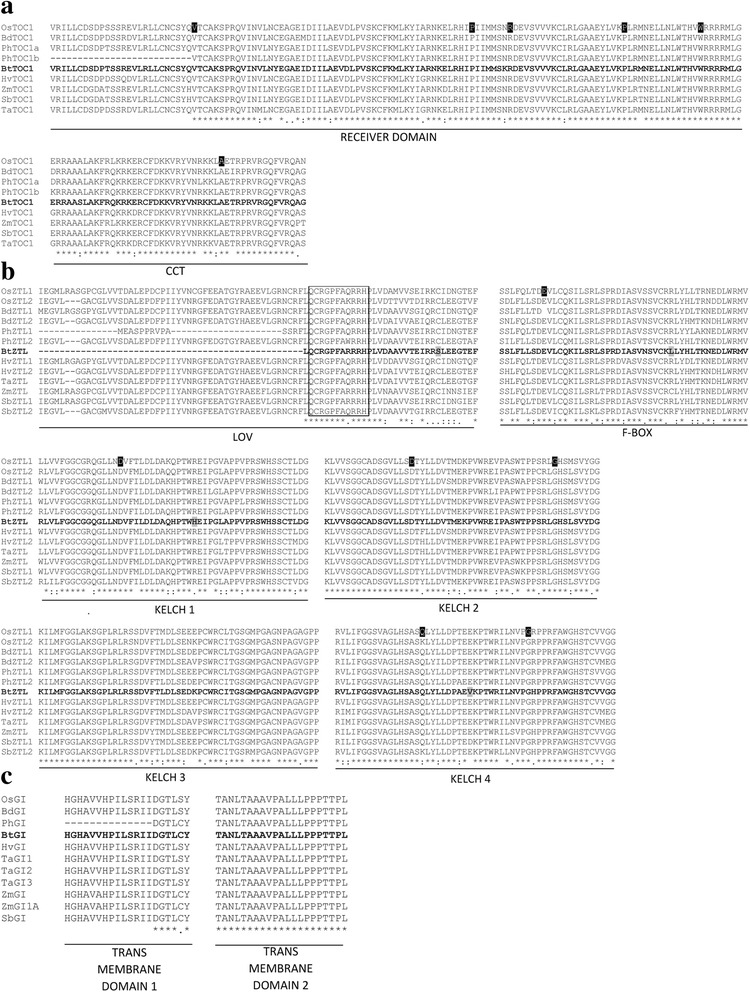
Multiple sequence alignment of *BtTOC1, BtZTL* and *BtGI* genes with homologous sequences from related monocots. Black highlighted amino acids are functionally important as evidenced by mutational analyses. **a** Detailed sequence characterization of *BtTOC1* and identification of receiver and CCT domains. Sequences used are: OsTOC1: Os02g40510.1, BdTOC1: Bradi3g48880, PhTOC1a: PH01003618G0130, PhTOC1b: PH01000345G0790, BtTOC1: KY249524, HvTOC1: AEW48242.1, ZmTOC1: ADX60159.1, SbTOC1: SORBI_004G216700, TaTOC1: AMK48975.1 **b** Detailed sequence characterization of *BtZTL* and identification of Light Oxygen Voltage (LOV), F-box domain and four Kelch repeats. Sequences used are: OsZTL1: Os06g47890.2, OsZTL2: Os02g05700.2, BdZTL1: Bradi1g33610.2, BdZTL2: Bradi3g04040.2, PhZTL1: PH01007024G0030, PhZTL2: PH01000836G0340, BtZTL: MF983715, HvZTL1: HV273830G00010, HvZTL2: HV158755G00020, TaZTL: ABR14627.1, ZmZTL: GRMZM2G113244, SbZTL1: Sobic.010G243900.1, SbZTL2: Sobic.004G042200.2. **c** Detailed sequence characterization of *BtGI* and identification of two characteristic trans membrane domains. Sequences used are: OsGI: Os01g08700.2, BdGI: Bradi2g05226.1, PhGI: PH01002142G0290, BtGI: MF983716, HvGI: AAW66945.1, TaGI1: AAQ11738.1, TaGI2: AAT79486.1, TaGI3: AAT79487.1, ZmGI: ABZ81992.1, ZmGI1A: DAA06172.1, SbGI: Sobic.003G040900.3

Single copies of the important circadian clock genes *LHY*, *TOC1*, *ZTL* and *GI* were identified in *B. tulda* (MF983713, KY249524, MF983715, MF983716). In order to obtain these genes and/or coding sequences, degenerate as well as gene specific primers were used for PCR amplification and subsequent sequencing (Additional file 1: Table S1). These sequences were used for BLAST analysis to identify their homologs in other monocot genomes. The best BLASTP hits obtained for *BtLHY*, *BtTOC1*, *BtZTL* and *BtGI* query sequences were *Oryza brachyantha* XP_006659145.1, *O. sativa* BAD38854.1, *Thyridolepis multiculmis* AML79118.1 and *Setaria italica* XP_004968438.1 having 78%, 85%, 93% and 94% sequence identities, respectively (Table 1). The translated *B. tulda* amino acid sequences were studied to identify the domains characteristics for these proteins. Indeed, the BtTOC1 sequence revealed the 127 amino acid receiver domain in the N-terminal end and 47 amino acid CCT domain in the C-terminal end (Fig. 2a). Like other ZTL proteins, BtZTL possessed N-terminal photo sensory light oxygen voltage (LOV) domain, F-box domain at the middle, and 4 kelch repeats at the C-terminal end (Fig. 2b). The other identified clock gene *BtGI* contained a transmembrane domain in the N-terminal region (Fig. 2c).

**Table 1:** Identification of *B. tulda* homologous sequences of circadian clock, clock integrator and pathway integrator genes

Genes	*B. tulda* query sequences	Best *O. sativa* hits	Identity (%)	Query cover (%)	E value	Best *B. distachyon* hits	Identity (%)	Query cover (%)	E value	Best *P. heterocycla* hits	Identity (%)	Query cover (%)	E value	Best hits in NCBI non-redundant database	Identity (%)	Query cover (%)	E value
*LATE ELONGATED HYPOCOTYL* (*LHY*)	MF983713	Os08g06110	78.31	100	0	Bradi3g16515	73	100	0	PH01001283G0510	77	100	0	*Oryza brachyantha* (XP_006659145.1)	78	100	0
*TIMING OF CAB EXPRESSION 1* (*TOC1*)	KY249524	Os02g40510	84	100	0	Bradi3g48880	80	95.21	0	PH01003618G0130	93	100	0	*O. sativa* (BAD38854.1)	85	100	0
*ZEITLUPE* (*ZTL*)	MF983715	Os02g05700	90.28	100	0	Bradi3g04040	89	100	0	PH01000114G1110	93	100	0	*Thyridolepis multiculmis* (AML79118.1)	93	100	0
*GIGANTEA* (*GI*)	MF983716	Os01g08700	91	100	0	Bradi2g05226	88	100	0	PH01002142G0290	70	100	E^− 129^	*Setaria italica* (XP_004968438.1)	94	100	0
*CONSTANS A* (*COA*)	KY249523	Os06g16370	59	100	1.00E^− 148^	Bradi1g43670	74.08	100	4.10E^−120^	PH01005551G0030	51	97.64	3.00E^−94^	*Oryza. rufipogon* (AFK31610.1)	78	100	0
*CONSTANS B* (*COB*)	MF983714	Os09g06464	71.06	100	1.00E^− 141^	Bradi3g56260	34.04	92.66	4.80E^−17^	PH01000048G0270	68	100	E^−117^	*Hordeum vulgare* (AAM74066.1)	70	100	2.00E^−138^
*FLOWERING LOCUS T* (*FT*)	KT003820	Os06g06320	89.71	98.31	1.00E^−118^	Bradi1g48830	93	87.64	1.60E^−106^	PH01002288G0050	62	85.95	7.00E^−52^	*Phyllostachys meyeri* (BAI49899.1)	94	100	5.00E^−122^
	KT003821	Os06g06320	89.71	98.31	1.00E^−118^	Bradi1g48830	93	87.64	1.60E^−106^	PH01002288G0050	62	85.95	7.00E^−52^	*P. meyeri* (BAI49899.1)	94	100	5.00E^−122^
	KU726232	Os06g06320	87.43	98.31	1.00E^−115^	Bradi1g48830	89	99.43	2.00E^−116^	PH01002288G0050	62	94.94	5.00E^−58^	*P. meyeri* (BAI49900.1)	92	100	4.00E^−118^
	KX290774	Os06g06320	88	98.31	1.00E^−116^	Bradi1g48830	90	99.43	2.30E^−117^	PH01002288G0050	62	94.94	8.00E^−59^	*P. meyeri* (BAI49900.1)	92	100	7.00E^− 119^

The BLAST-P analyses was done against the reference monocot genome *Oryza sativa*, phylogenetically close *Brachypodium distachyon*, temperate bamboo *Phyllostachys heterocycla* and NCBI non-redundant database using *B. tulda* amino acid sequences as quarries. Only the top BLAST hit sequences are reported along with the respective identities (%), E values and coverage of the query sequences against the obtained hit sequences (%)

### Molecular identification, sequence characterization and phylogenetic analyses of *BtCOA* and *BtCOB* genes

**Fig. 3:**
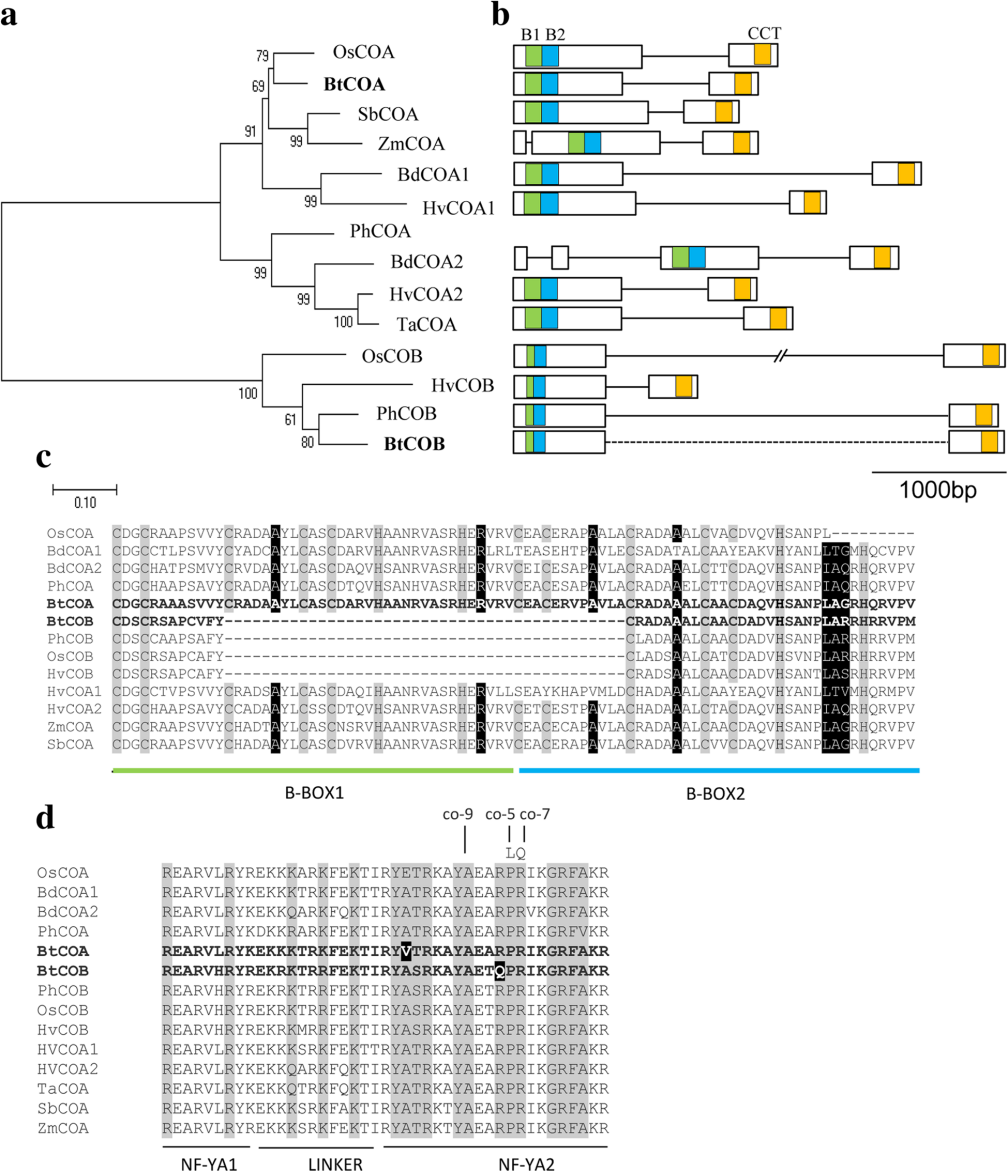
Phylogenetic and sequence characterization of *BtCOA* and *BtCOB* genes. **a** Phylogenetic comparison of *BtCOA* and *BtCOB* coding sequences with homologous sequences in related monocot species. The Neighbour Joining (NJ) tree was developed by Mega 7.0 using default parameters and bootstrap value 1000. **b** Predicted exon-intron structures of *BtCOA*, *BtCOB* genes and comparison with other monocot genes. Exons are marked as rectangles and introns as solid lines. **c** Multiple sequence alignment of the B-box domains of *BtCOA* and *BtCOB* protein sequences, which indicates presence of two full length B-boxes in *BtCOA*, while it is truncated in *BtCOB.* The characteristic C and H residues of B-box domains are highlighted in grey. Black highlighted amino acids are functionally important as evidenced by mutational analyses. **d** Sequence comparison of CCT domains between *BtCOA*, *BtCOB* and other related monocot members. Amino acids conserved for HAP3 and HAP5 binding are highlighted in grey. Amino acids not conserved in *B. tulda* are highlighted in black. NF-YA1 interacts with HAP3 and NF-YA2 interacts with CCAAT DNA sequences. Sequences used are: *OsCOA*: Os06g16370.1, *BdCOA1*: Bradi1g43670.1, *BdCOA2*: Bradi3g56260.1, *PhCOA*: PH01005551G0030, *BtCOA*: KY249523, *HvCOA1*: AF490467.1, *HvCOA2*: AF490469.1, *ZmCOA*: GRMZM2G405368, *SbCOA*: Sobic.010G115800.1, *OsCOB*: Os09g06464.1, *PhCOB*: PH01000048G0270, *BtCOB*: MF983714, *HvCOB*: AF490473.1

*CONSTANS* (*CO*) is the circadian clock response integrator gene, which is a member of the B-box family [33]. Single copy *BtCOA* and *BtCOB* genes were amplified from gDNA and cDNA libraries, sequenced and analysed (KY249523, MF983714). The BtCOA protein sequence was most identical to *Oryza rufipogon* sequence (AFK31610.1) having 78% identity, while the highest identity (70%) of BtCOB was detected against barley (AAM74066.1, Table 1). Phylogenetic analyses based on the amino acid sequences revealed a clear split of *BtCOA* and *BtCOB* genes into two different clades (Fig. 3a). While *BtCOA* was more closely related to rice *OsCOA* than the temperate bamboo *Phyllostachys PhCOA*, *BtCOB* clustered with *PhCOB*. This indicated that with respect to gene sequences the two *BtCO*s were quite divergent. Prediction of gene models indicated that like other characterized *CO* sequences, *BtCOA* and *BtCOB* contained two exons and one intron each. The intron lengths of *COB* varied across species, while in *COA* it was more conserved (Fig. 3b). The translated BtCOA and BtCOB proteins were of 382 and 327 amino acids in length. Protein sequence analyses revealed that both BtCOA and BtCOB contained two B-boxes at their N-terminal ends (B-box 1, B-box 2) having conserved C and H residues (Fig. 3c). An intact 43 amino acid long B-boxes 1 and 2 was obtained for BtCOA. In contrast, 25 amino acids of the C-terminal end of B-box 1 and 18 amino acids in the N-terminal part of the B-box 2 were truncated in BtCOB (Fig. 3b, c). In addition to the N-terminal B-box domain, BtCOA and BtCOB possessed a 43 amino acid DNA binding CCT domain in their C-terminal ends (Fig. 3d). In plants the CCT domain interacts with other DNA binding proteins such as HAP3 and HAP5 with the help of nine conserved amino acids [34]. While all these amino acids were conserved in BtCOA, Arg33 was changed to Gln33 in BtCOB (Fig. 3d).

### Molecular identification, sequence characterization, phylogenetic analyses of four *BtFT* genes

**Fig. 4:**
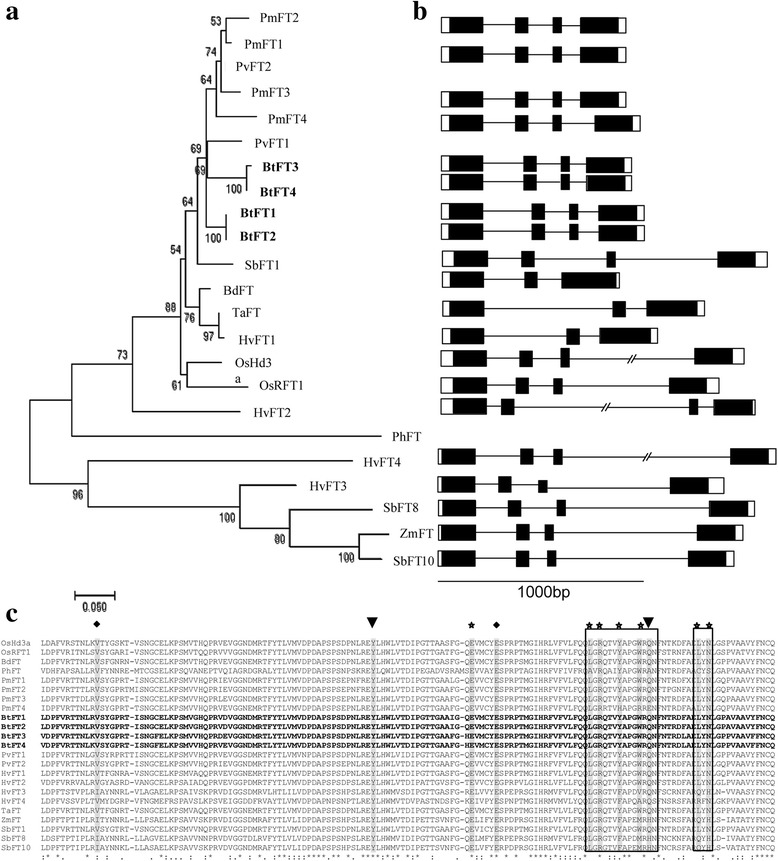
Phylogenetic and sequence characterization of four *BtFT* genes. **a** Phylogenetic comparison of *BtFT1, BtFT2, BtFT3* and *BtFT4* coding sequences with homologous sequences in related monocot species. The Neighbour Joining (NJ) tree was developed by Mega 7.0 using default parameters and bootstrap value 1000. **b** Predicted exon-intron structures of four *BtFT* genes and comparison with other monocot genes. Exons are marked as rectangles having PEBP domains marked in solid black boxes and introns as solid lines. **c** Sequence comparison of the PEBP domains of *BtFT* and other related monocot sequences. Two residues marked with arrow heads are characteristics for either *FT* or *TFL1* identity. Residues having important biological functions are marked in asterisks. Sequences used are: *OsHd3a*: Os06g06320.1, *OsRFT1*: Os06g06300.1, *BdFT*: Bradi1g48830.1, *PhFT*: PH01002288G0050, *PmFT1*: AB498760.1, *PmFT2*: AB240578.1, *PmFT3*: AB498761.1, *PmFT4*: AB498762.1, *BtFT1*: KT003820, *BtFT2*: KT003821, *BtFT3*: KU726232, *BtFT4*: KX290774, *PvFT1*: Guo et al. (2015), *PvFT2*: Guo et al. (2015), *HvFT1*: DQ100327, *HvFT2*: DQ297407.1, *HvFT3*: DQ411319, *HvFT4*: DQ411320, *TaFT*: DQ890162.1, *ZmFT*: EU241924, *SbFT1*: XP_002436509.1, *SbFT8*: XP_002456354.1, *SbFT10*: Sb09g025760

*Flowering locus T* (*FT*), a member of PEBP family, is one of the most important floral pathway integrator genes. In the present study, four alleles of *BtFT* genes were identified (Additional file 2: Figure S1, *BtFT1*: KT003820, *BtFT2*: KT003821, *BtFT3*: KU726232, *BtFT4*: KX290774). A homology search using translated coding sequences of the *BtFT1*, *2, 3* and *4* revealed very high identity (92-94%) with *FT* sequences of another bamboo *P. meyeri* (Table 1)*.* The four BtFT sequences were phylogenetically separated into two different clades (Fig. 4a). While *BtFT1* clustered with *BtFT2*, *BtFT3* clustered with *BtFT4*, indicating that the two groups of genes are distinct based on their sequences. This finding was also supported by their predicted exon-intron organization (Fig. 4b). Each of the four *BtFT* genes contained four exons and three introns. Exon 4 was the longest (233 bp), while exon 3 was the shortest (41 bp). Although the exon lengths were highly conserved among the 4 *BtFT* homologs, the length of intron 1 was longer in *BtFT1, 2* than that of *BtFT3, 4* (Fig. 4b). Each predicted BtFT protein was 178 amino acid long, having a PEBP domain that retained seven conserved amino acid residues and two C-terminal amino acid stretches, which are important for maintaining the floral inducing function. Incidentally, another PEBP member is *TERMINAL FLOWER1* (*TFL1*), which is a floral repressor and is highly similar in sequence to *FT*. Among the differences are two signature amino acids, Tyr85 and Gln140 present in FT (Fig. 4c), while His88 and Asp144 in TFL1 instead [35]. The present analysis confirmed that all the identified sequences are indeed *FT*, not *TFL1* (Fig. 4c).

### In silico study on the molecular interactions between individual BtFT and Os14-3-3 proteins

**Fig. 5:**
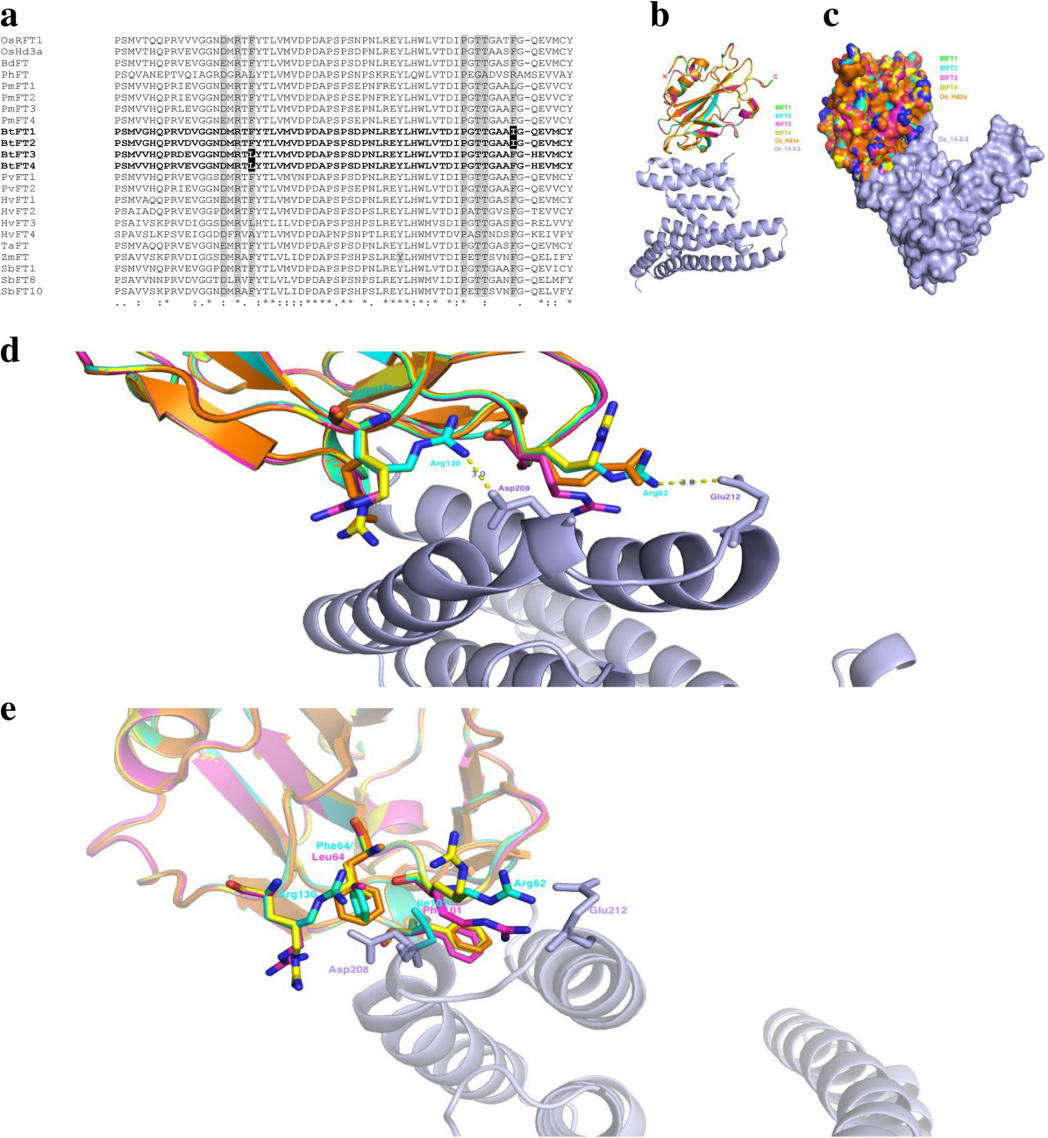
In silico study on the molecular interactions between individual BtFT and Os14-3-3 proteins. **a** Detailed sequence analysis of BtFT protein regions responsible for interacting with 14-3-3 protein. Seven amino acids conserved for 14-3-3 interaction are highlighted in grey. Amino acids not conserved in *B. tulda* are highlighted in black. Sequences used are: OsHd3a: Os06g06320.1, OsRFT1: Os06g06300.1, BdFT: Bradi1g48830.1, PhFT: PH01002288G0050, PmFT1: AB498760.1, PmFT2: AB240578.1, PmFT3: AB498761.1, PmFT4: AB498762.1, BtFT1: KT003820, BtFT2: KT003821, BtFT3: KU726232, BtFT4: KX290774, PvFT1: Guo et al. (2015), PvFT2: Guo et al. (2015), HvFT1: DQ100327, HvFT2: DQ297407.1, HvFT3: DQ411319, HvFT4: DQ411320, TaFT: DQ890162.1, ZmFT: EU241924, SbFT1: XP_002436509.1, SbFT8: XP_002456354.1, SbFT10: Sb09g025760. **b** Conserved interaction pattern between BtFT and 14-3-3. Given more than 86% homology with each other as well as rice counterpart Hd3a, all BtFT proteins (1-4) are almost perfectly superimposable to each other. Their interaction pattern with 14-3-3 also remains mostly conserved. **c** Surface analysis showing interdigitated interface between a pair of BtFT and 14-3-3. **d** Conserved salt bridge interactions between BtFT and 14-3-3. Asp208-Arg130 and Glu212-Arg62 salt bridges could be essential for BtFT’s interaction not only with 14-3-3 but also with FD. **e** Difference in hydrophobic cavity lining BtFT proteins. Subtle alterations e.g. Phe101 to Ile101in BtFT1, 2 and Phe64 to Leu64 in BtFT3, 4 might alter the specificity of BtFT1,2/3,4 interaction with 14-3-3

The rice FT homologue Hd3a interacts with 14-3-3 proteins at the shoot apical meristem (SAM) to form the Hd3a-14-3-3 complex, which is translocated to the nucleus to interact with rice FD1, a bZIP transcription factor [36]. The resulting “florigen activation complex” (FAC), promotes the conversion of the SAM to an inflorescence meristem [36]. Out of seven conserved amino acids located within the PEBP domain of FT that contribute to the direct interaction between FT and 14-3-3 (Fig. 5a), two substitutions, from Phe101 to Ile101 in BtFT1, 2 and Phe64 to Leu64 in BtFT3, 4 were observed. In silico protein-protein interaction analyses were conducted to understand the overall interaction efficiency between individual BtFT and 14-3-3 sequences and to detect whether these changes affect the interaction. Since no crystal structures were available for BtFT proteins and no sequence or structure of Bt14-3-3, the interaction between BtFT and Os14-3-3 pairs were investigated. Homology models of BtFT1, 2, 3 and 4 were developed, and these were 86-88% identical to their rice homologue OsHd3a. Given the profound homologies among all BtFT alleles, their interaction with Os14-3-3 remained mostly conserved (Fig. 5b), with interaction interface remaining interdigitated (Fig. 5c). Similar to OsHd3a-Os14-3-3 interaction [36], BtFT1, 2, 3, 4 and Os14-3-3 interaction interface consisted of a hydrophobic cavity as well as an in-between acidic lobe (Asp208 and Glu212 of 14-3-3), interacting with Arg130 and Arg62 of BtFT1, 2, 3 and 4 (Fig. 5d) through conserved salt-bridge interactions. These interactions are essential not only for FT binding with 14-3-3 but also with FD. In contrast to OsHd3a sequence (Phe66 and Phe103), Leu64 was present in BtFT3, 4 and Ile101 was present in BtFT1, 2, respectively. In BtFT1, 2, Phe64 stabilized the hydrophobic interaction with Ile204 of Os14-3-3, similar to the OsHd3a interaction. In BtFT1, 2, Ile101 made hydrophobic contact with Phe200 of Os14-3-3, in BtFT3, 4, but there was a possibility of a stacking interaction between Phe101 and Phe200 of Os14-3-3, similar to Hd3a. Although the hydrophobic interactions (Fig. 5d) were subtly different in BtFT1, 2 and BtFT3, 4 compared to OsHd3a-Os14-3-3 interactions, such changes might influence the specificity of BtFT interactions with 14-3-3.

### Tissue specific expression analyses of circadian clock, *CO* and *FT* genes

**Fig. 6:**
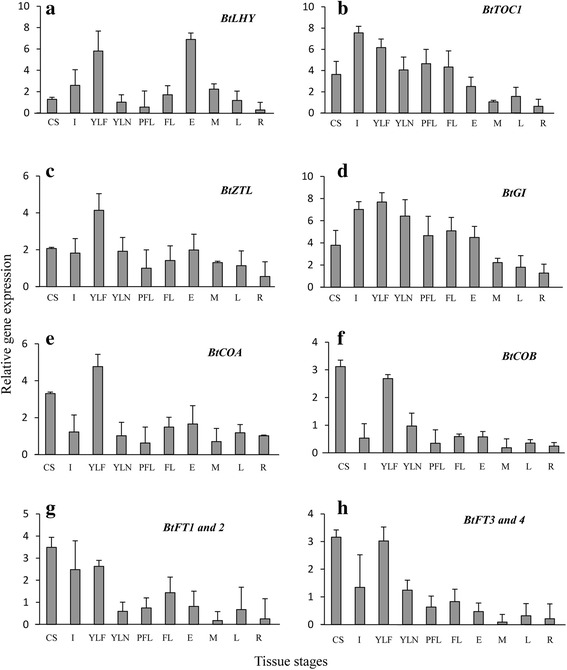
Study of relative gene expression levels in ten tissue stages of *B. tulda.* **a-h** spatial gene expression levels of *BtLHY*; *BtTOC1*; *BtZTL; BtGI; BtCOA*; *BtCOB*; *BtFT1, 2* and *BtFT3, 4.* Transcript expression of *eIF4α* was used to normalize expression data of the targeted flowering genes. The relative fold change was calculated by 2^-∆∆CT^ method using the expression level observed in rhizome as the calibrator. Each bar represents mean of three biological replicates ± SE

The transcriptional expression of the circadian clock (*BtLHY, BtTOC1, BtZTL, BtGI*)*,* circadian clock integrator (*BtCOA, BtCOB*) and floral pathway integrator (*BtFT1, BtFT2, BtFT3* and *BtFT4*) genes were investigated in ten selected flowering and non-flowering tissue stages to understand their possible role in flowering. Higher transcript abundance of all these genes was detected in young leaves isolated from the flowering culm (YLF) than that of the non-flowering culm (YLN). However, when the expression levels were compared among ten tissues, the highest expression of *BtLHY* and *BtTOC1* was obtained in early stage inflorescence bud and internodal tissues (Fig. 6a, b), while it was YLF in case of *BtZTL* and *BtGI* (Fig. 6c, d). In the case of *BtCOA* and *BtCOB*, higher transcriptional expression was detected in YLF and culm sheath (CS) respectively, while the expression level was consistently low in all other eight tissues. The expression of *BtCOA* was much higher in YLF than CS, although such a clear distinction in expression levels was absent in *BtCOB* (Fig. 6e, f). This is an important indication of the possible involvement of *BtCOA* in floral induction, because YLF is biologically associated with the floral induction while CS is mostly vegetative in nature. Such distinctions in expression patterns between flowering and non-flowering tissue stages were not observed for the two groups of *BtFT* genes that were suggested by the phylogenetic analysis (Fig. 6g, h). The highest expression of all of the four homologs was observed in CS. However, in case of *BtFT3*, *4* the expression was also quite high in YLF, which was not the case for *BtFT1*, *2*.

### Study on the diurnal expression patterns of circadian clock genes

**Fig. 7:**
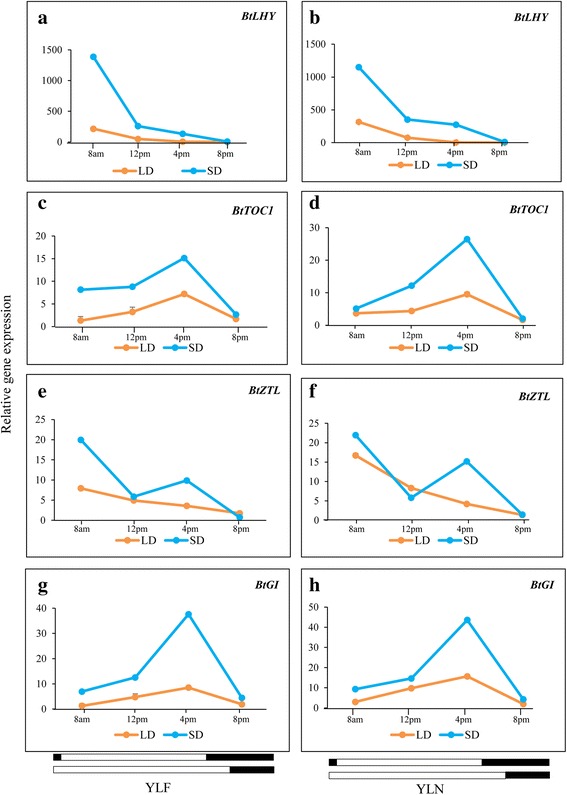
Comparison of diurnal expressions of circadian clock genes in YLF and YLN during SD and LD. **a**, **b** *BtLHY*, **c**, **d** *BtTOC1*, **e**, **f** *BtZTL* and **g**, **h** *BtGI*. Transcript expression of *eIF4α* was used to normalize expression data of targeted flowering genes in different tissues. The relative fold change was calculated by 2^-∆∆CT^ method using the expression data in rhizome as calibrator and is plotted using two Y axis. Each data point in the line graph represents mean of three biological replicates ± SE in case of LD and one biological replicate in case of SD

Although tissue specific expression patterns can provide important clues about gene functionality, the majority of genes studied here need to follow a circadian rhythm in order to perform their developmental role in the plant. Therefore, the diurnal expression patterns of the circadian clock genes (*BtLHY*, *BtTOC1*, *BtZTL* and *BtGI*) were studied at four different time points (morning: 8 am, noon: 12 pm, afternoon: 4 pm, night: 8 pm) under the short-day (11 h light) and long-day (14 h light) conditions of the natural habitat of the plants. Two sets of leaf tissues were selected for this study. The leaves collected from a flowering culm (YLF) were selected due to their anticipated involvement in floral induction, which is supported by obtaining higher level of expression of the clock genes compared to the other leaf tissues. On the contrary, the leaves from a non-flowering culm (YLN) were selected as the comparable tissue representing the non-inductive stage. In general, the transcript abundance of all these genes was detected at higher levels under SD than LD, both for YLF and YLN (Fig. 7a-h). The diurnal expression patterns of *BtTOC1* and *BtGI* attained a peak in the afternoon while *BtLHY* and *BtZTL* transcripts were abundant in the morning, followed by a gradual decrease under SD (Fig. 7a-h).

### Study on the diurnal expression patterns of *BtCO* and *BtFT* genes

**Fig. 8:**
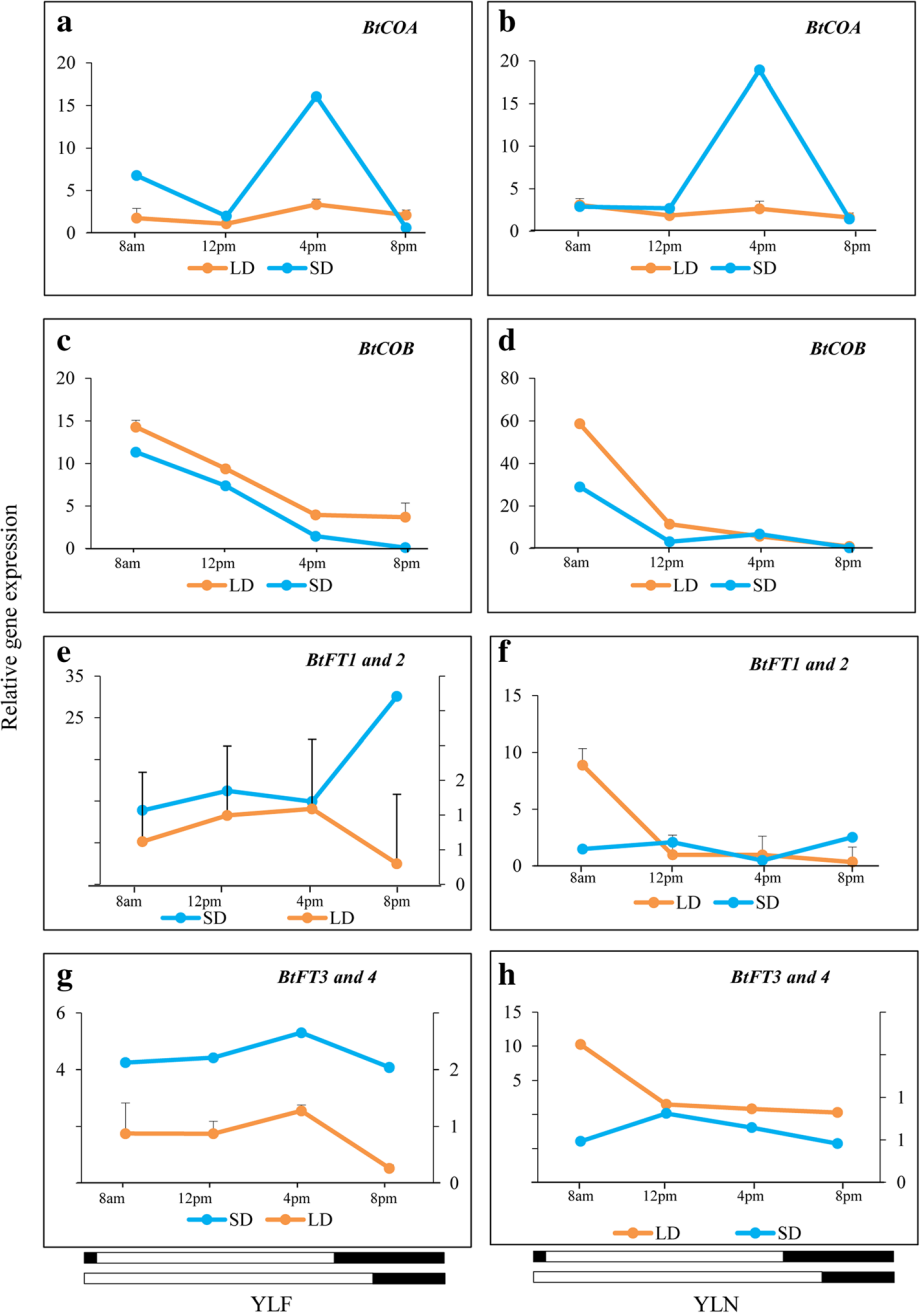
Comparison of diurnal expressions of *BtCO* and *BtFT* homologs in YLF and YLN during SD and LD. **a**, **b** *BtCOA*, **c**, **d** *BtCOB*, **e**, **f** *BtFT1, 2* and **g**, **h** *BtFT3, 4*. Transcript expression of *eIF4α* was used to normalize expression data of the targeted flowering genes in different tissues. The relative fold change was calculated by 2^-∆∆CT^ method using the expression data in rhizome as calibrator and is plotted using two Y axis. Each data point in the line graph represents mean of three biological replicates ± SE in case of LD and one biological replicate in case of SD

The circadian oscillations acquired by the circadian clock genes are transmitted to *CO*, which eventually interacts with *FT* to induce flowering. Therefore, the circadian rhythm of *CO* should be followed by *FT* in order to perform their assigned biological functions. In bamboo, the situation was not straightforward since multiple *CO* and *FT* gene copies/alleles were present. Therefore, diurnal expression of two *BtCO* and four *BtFT* homologs were measured in YLF and YLN under SD and LD conditions. Similar to the clock genes, the expression of *BtCOA* was higher in both YLF and YLN under SD than LD (Fig. 8a, b). In contrast, the opposite trend was observed for *BtCOB*, the homolog of which acts as a floral repressor in rice. The diurnal expression pattern of *BtCOA* reached a peak in the afternoon followed by a sudden decrease. In contrast, the maximum expression level of *BtCOB* was observed in the morning and gradually decreased throughout the day (Fig. 8a, b). The diurnal expression patterns of *BtCOA* and *BtCOB* were compared to that of four *BtFT* alleles. Similar to *BtCOA*, the diurnal expression pattern of *BtFT3, 4* revealed its highest expression in the afternoon in both SD and LD condition in YLF, but not in YLN. In contrast, the diurnal expression pattern of *BtFT1, 2* did not follow that of *BtCOA* or *BtCOB*.

## Discussion

Molecular studies on bamboo flowering are limited and the primary reasons are the unavailability of sufficient reproductive tissues and undefined developmental stages [17]. The possible alternatives, such as use of annual flowering bamboo (e.g., *Indocalamus wightianus, Ochlandra sp*.) or use of in vitro induced flowering tissues [37] are not credible since they either lack the extended vegetative phase or the plants are grown under artificial conditions and therefore regulation of the genes might be different. Consequently, apart from a few exceptions [20], the majority of studies have relied on field-grown flowering plants, undertook de novo transcriptome sequencing of floral tissues and annotated short ESTs based on BLAST based sequence homology [20–27]. All these studies yielded important but partial understanding of the genes and their regulation, as they do not provide full-length gene sequences or detailed expression profiles. In the absence of those data, collective characterization of genes involved in a particular flowering pathway remains elusive in bamboo.

### Important diurnally regulated circadian clock genes are identified in *B. tulda*

Plant circadian rhythms in response to light, are regulated by a series of interconnected transcriptional and translational loops of clock related genes. The roles of these genes have been extensively studied in reference plants, *A. thaliana* and rice, which are mostly annual [7, 13]. In rice *OsLHY*, *OsTOC1, OsZTL* and *Os*GI are the key components of the core feedback loop of the circadian clock [38–41]. The *OsLHY* is up regulated in the morning via red light [42]. This elevated *OsLHY* transcript supressed the expression of *OsTOC1* in the morning [39, 43], but by evening *OsTOC1* regained transcriptional peak. Eventually OsTOC1 upregulated *OsLHY* and simultaneously suppressed OsGI. On the other hand, the upregulation of OsGI in the evening was caused by the blue light mediated degradation of OsTOC1 by OsZTL [38, 44]. In our study the identified *B. tulda* gene homologs were highly identical to sequences obtained from other monocots including *Phyllostachys*. The overall diurnal rhythms of *BtLHY*, *BtTOC1* and *BtGI*, but not *BtZTL*, were comparable to that of rice [39, 44–46]. The *OsZTL* showed a unimodal expression peak in the morning under SD, but was bimodal (morning and afternoon) under LD [39, 45]. However, this trend was reversed in *B. tulda*, where the observed diurnal peak was unimodal (only morning) under LD and bimodal (morning and afternoon) under SD. This could be a significant clue for future studies as because it is established that the function of *ZTL* is primarily flower specific, while the other circadian clock genes such as *LHY* and *TOC1* perform pleiotropic functions including leaf movement, maintenance of hypocotyl length, expression of antenna protein, cell elongation and UV-B protection [47–49].

### Distinct sequence and expression divergence observed for the two types of *CONSTANS* genes identified

A large number of *CONSTANS like* genes (*COL*s) are present in plants. For example, there are 17 *COL*s in *A. thaliana*, 16 in rice, and 26 in soybean [33, 50]. Depending on the number of B-boxes present, all these *COL*s can be grouped into four different clusters, which are indicated as I, II, III and IV [33]. The members of group I *COL*s primarily act as floral regulators and may act either as floral activators or repressors [51–54]. In *B. tulda* two *CO* genes have been identified, *BtCOA* and *BtCOB*, which are the members of the group I cluster. The B-box domain organization (two intact B-boxes in *BtCOA* vs. truncated B-boxes in *BtCOB*) and tissue specific expression patterns (*BtCOA* expression is high in YLF, while *BtCOB* is highest in CS) indicate that *BtCOA* is possibly involved in photoperiodic regulation of flowering, while *BtCOB* is not. This was further supported by the diurnal circadian rhythm. *BtCOA* exhibited a transcript expression peak in the afternoon, which is similar to the flower inductive rice *OsCOA* homolog *HEADING DATE1* [55, 56]. On the contrary *BtCOB* demonstrated an expression peak in the morning. The rice *COB* homolog *OsCO3*, which is a negative regulator of *OsHd3a,* also demonstrated a similar diurnal rhythm [57]. All this evidence suggests that *BtCOA* contains biologically important sequence elements and characteristic diurnal expression patterns, which were not observed in *BtCOB.* Further studies are required to verify whether additional *CO like* genes exist in bamboo and, if so, how they contribute to flower induction.

### High sequence similarity, but differential diurnal regulation indicates possible functional divergence of four *BtFT* homologs

*FT* is a member of the PEBP family and is present in multiple copies in different plant species [58–68]. In *B. tulda* four *FT* alleles have been identified, which are more than 98% similar in terms of their amino acid sequences. The individual amino acid differences in the four *BtFT* sequences, particularly in context to 14-3-3 interactions, were carefully considered to predict their possible influence on *FT* functioning. Phylogenetic as well as in silico interaction analyses clearly indicated that *BtFT1* was more homologous to *BtFT2*, while *BtFT3* was closer to *BtFT4*. Though most essential salt bridge interactions between 14-3-3-BtFT pairs, Asp208-Arg130 and Glu212-Arg62 were conserved, there was little change in the composition of the hydrophobic cavity lining BtFT. Such a subtle change in hydrophobicity, though apparently not drastic, might influence the specificity of BtFT and 14-3-3 interactions.

There exists wide diversity with respect to the roles of *FT* gene copies in flowering. In poplar, expression divergence leading to distinct subfunctionalization has been noticed between the two *FT* genes [65]. While *PtFT1* is primarily responsible for inducing reproductive development, *PtFT2* is involved in the vegetative growth of the plant. Similarly, expression diversification of the two *FT* genes was also reported in the temperate bamboo *P. violascens* [68]. *PvFT1* is expressed in leaves and induces flowering, while *PvFT2* possibly plays an important role in floral organ differentiation. Since flowering is an environmentally regulated biological process, the regulation of genes related to flowering is likely to be different in temperate and tropical bamboos. Circadian oscillation of *BtFT3* and *4* in YLF revealed highest expression in the afternoon, while no such pattern was observed in case of YLN under either SD or LD conditions. In *A. thaliana*, barley and soybean the diurnal expression rhythm of *FT* showed a transcriptional peak in the afternoon [69, 70], while for rice it was in the morning [44]. The diurnal expression pattern of *BtFT1* and *2* in both YLF and YLN was quite divergent to that of *BtFT3* and *4*. The expression divergence of *BtFT* genes might have been caused by changes in the promoter regions since such observations have been made in rice and *Brassica* [71, 72]. Therefore, native bamboo gene promoters should be sequenced in order to understand the expression regulation of these genes.

### Existence of *CO-FT* regulon in bamboo

For the induction of flowering, the specific diurnal rhythm of *CO* has to be followed by *FT*. It has been observed in many plants such as soybean and rice that out of multiple copies of *CO* and *FT* genes, only a few candidates follow the diurnal expression pattern necessary for flower induction [70, 73]. In *Glycine max*, among the 28 *CO* and 11 *FT* like genes, the diurnal expression pattern of *GmCOL5* and *GmCOL13* synchronized with 6 *GmFT* genes [70]. In poplar the co-expression of *PtCO2* and *PtFT1* gene pairs controls the timing of flowering and is known as the CO-FT regulon [74]. In bamboo four *FT* genes have been identified in *P. meyeri* and their tissue specific expression patterns have been studied [67]. However, no information could be obtained on any bamboo *CO* homologs and their expression patterns in different tissues and diurnal conditions. In the absence of such data the possible existence of *CO-FT* regulon in bamboo flowering could not be tested. Therefore, the synchronization of the diurnal expression patterns of *BtCO* and *BtFT* gene copies were investigated. Indeed, the diurnal oscillation of *BtCOA* was followed by *BtFT3* and *4* in YLF, but not in YLN suggesting the possible existence of *CO-FT* regulon in bamboo. Further studies are required to confirm the functional significance of this finding in terms of regulation of flowering in bamboo.

## Conclusion

Sequence comparison, phylogeny, and expression analyses of the studied genes indicate existence of an active photoperiodic pathway in bamboo. The findings also indicate that an increase in gene copy numbers and expression divergences of *CO* and *FT* play an important role in photoperiodic regulation of flowering in bamboo. Involvement of many more additional factors such as physiological maturity [75], micro RNAs [76] or RNA splicing [77] may ultimately determine the timing of flowering. Further studies are required to characterize many of the genes identified here by loss-of-function or overexpression analyses to understand their functional role in bamboo flowering. Taken together, the present findings would not only be useful for future research on bamboo but also for the non-reference plants that remain neglected.

## Methods

### Identification and collection of appropriate flowering and vegetative tissues in *B. tulda*

Floral tissue samples of *B. tulda* were collected from sporadic flowering events that happened at Shyamnagar (22.83° N, 88.40° E) and Bandel (22.93° N, 88.38° E), West Bengal, India during April, 2013 to July, 2017. Voucher specimen were submitted to the Botanical Survey of India (B.S.I), Shibpur (deposition nos.-56A, 56B, 57A, 57B, 58A. 58B, 59A, 59B, 59C dated 05.06.2015). Tissues from diverse vegetative and floral developmental stages were snap frozen in liquid nitrogen in the field, transported to the laboratory and stored in the -80 °C freezer. Three biological replicates were collected for each tissue stage. Vegetative tissues selected for tissue specific expression analyses were leaf from both flowering and non-flowering branches of a flowering culm, leaf from non-flowering culm, flag leaf, culm sheath, rhizome, root and internodal region (Fig. 1). Three defined floral tissues stages such as early, middle and late developmental stages were selected based on the histological observations of the developing floral primordia [17]. For diurnal analyses, leaf tissues were selected from non-flowering culm and non-flowering branches of flowering culm. Tissues were collected from naturally grown plants at four different time points of a day- morning (8 am), noon (12 pm), afternoon (4 pm) and night (8 pm) for both long-day (LD, 14 h light exposure, sunrise at 4:30 am and sunset at 6:30 pm) and short-day (SD, 11 h light exposure, sunrise at 6 am and sunset at 5 pm). LD experiments were conducted using three biological replicates, while only one replicate was available for SD analyses.

### Isolation of nucleic acids and preparation of cDNA libraries

Genomic DNA was isolated from the young, healthy leaves using DNeasy Plant Mini Kit (Qiagen, Germany). Total RNA was extracted from the selected tissues using a combination of Trizol (Invitrogen, USA) and RNAeasy Plant Mini Kit (Qiagen, Germany) [78, 79]. DNase I (Thermo Scientific, USA) was added to avoid any genomic DNA contamination. Quality and quantity of the isolated samples were determined in a BioSpectrometer (Eppendorf, Germany) and agarose-formamide gel elctrophoresis. Around 1 μg total RNA was used for cDNA synthesis using Verso cDNA Synthesis Kit (Thermo Scientific) following manufacturer’s protocol. 2 μl of 1/20th diluted cDNA sample was used for real time RT-qPCR analyses.

### Primer designing, PCR amplification, cloning and sequencing of homologous genes

Gene specific degenerate primers were designed by aligning multiple sequences retrieved from related close monocot genomes (Additional file 1: Table S1). Coding sequences were multiple aligned in MUSCLE and gene specific primers were designed by using Primer3 program. PCR amplification was done using high fidelity Phusion Taq DNA polymerase (Thermo Scientific). Amplified bands of desired molecular weight were eluted from agarose gel by using GeneJET gel elution kit (Thermo Scientific) and cloned into TA vector (pGEM®-T Easy Vector Systems, Promega, USA) or blunt end vector (pJET PCR cloning kit, Thermo Scientific) following the instructions of the manufacturers. Positively transformed colonies were selected on blue-white selection and/or ampicillin medium and plasmids were purified using plasmid isolation kit (GeneJET Plasmid Miniprep Kit, Thermo Scientific). Sequencing was done by Sanger’s method, trimmed to remove vector sequences, assembled by CAP3 [80] and used for all further bioinformatics analyses. Comparisons with other known sequences revealed identification of full length *BtTOC1; BtCOA; BtFT1, 2, 3, 4* genes. Although, the other four genes (*BtLHY*, *BtZTL*, *BtGI* and *BtCOB*) could be partially sequenced, biologically important domain regions were mostly present in the sequenced regions. All sequence data were deposited at NCBI (http://www.ncbi.nlm.nih.gov/) *BtFT1* (KT003820), *BtFT2* (KT003821), *BtFT3* (KU726232), *BtFT4* (KX290774), *BtCOA* (KY249523), *BtCOB* (MF983714), *BtTOC1* (KY249524), *BtLHY* (MF983713), *BtZTL* (MF983715), *BtGI* (MF983716).

### Sequence data and phylogenetic analyses

The amino acid sequences of the identified *B. tulda* genes were aligned with other related sequences using the Clustal W program. The sequences were compared to that of available sequences from related monocots genomes such as *Oryza sativa, Phyllostachys meyeri*, *P. heterocycla*, *P. violascens, Brachypodium distachyon, Sorghum bicolor*, *Hordeum vulgare*, *Zea mays* and *Triticum aestivum*. The phylogenetic tree was constructed by the NJ method with Mega 7 software [81]. Bootstrap analysis with values for 1000 replicates was conducted to estimate nodal support. All available literatures were consulted to identify specific amino acid residues within the target genes that are involved in significant biological functions.

### In silico study on the molecular interactions between individual BtFT and Os14-3-3 proteins

Due to unavailability of crystal structures of BtFT1-4 and sequence/and structure of Bt-14-3-3, interaction between the BtFT-14-3-3 pairs was investigated, keeping 14-3-3 structural coordinates [36] constant from rice Os14-3-3. Homology models of BtFT1-4, which were 86-88% identical to their rice homologue OsHd3a, were built using the web version of MODELLER [82]. Interaction analyses were carried out using PyMOL.

### Gene expression analyses by real time RT-qPCR method

Gene specific primers were designed from the coding sequences of the targeted genes to measure their transcriptional expression level by real time RT-qPCR analyses (Additional file 1: Table S1). Sequences of four *BtFT* gene alleles were so similar that it was rather impossible to design individual primers for each. Therefore, one pair of primers was designed for *BtFT1* and *2*, while another was designed for *BtFT3* and *4* and that too were designed only based on one nucleotide sequence divergence at the 3′ end. The identity of the amplified gene products was confirmed by sequencing the amplified PCR products. SsoAdvanced™ Universal SYBR^®^ Green Supermix (Bio-Rad, USA) was used to measure the expression level of the targeted genes in CFX connect real-time PCR detection system (Bio Rad). The amplification conditions were 30 s at 95 °C, 40 cycles of 10 s at 94 °C and 40 s at 55 or 64 °C. A standard dissociation curve analyses was conducted to confirm the absence of any primer dimers in the amplified products. Data were normalized using *eIF4α* as the reference gene and relative fold change in gene expression was estimated following the 2^-ΔΔCt^ method [83]. In a comprehensive study we have shown that *elF4α* is one of the most stable reference genes in *B. tulda* (data unpublished), therefore was used for data normalization in the current study.

## Additional files

Additional file 1: **Table S1**. A summary of the oligonucleotides used in this study for different purposes. (DOC 90 kb)

Additional file 2: **Figure S1**. Two PCR amplified bands using degenerate primers specific for *FLOWERING LOCUS T* (*FT*). (TIFF 472 kb)

## Abbreviations

BLAST: Basic Local Alignment Search Tool
CCT: CONSTANS, CONSTANS -like, TIMING OF CAB EXPRESSION 1
COA: CONSTANS A
COB: CONSTANS B
COLs: CONSTANS like genes
CS: Culm sheath
DNP: Day neutral plant
E: Early staged inflorescence bud
eIF4α: Eukaryotic initiation factor 4α
ESTs: Expressed sequence tags
FL: Flag leaf
FLR: Floret
FT: FLOWERING LOCUS T
GA_3_: Gibberellic acid 3
GI: GIGANTEA
GL: Glume
HAP: Heme activator protein
IN: Inter node
L: Late staged inflorescence bud
LDP: Long-day plant
LHY: LATE ELONGATED HYPOCOTYL
LM: Lemma
LOV: Light oxygen voltage
M: Middle staged inflorescence bud
PEBP: Phosphatidyl ethanolamine binding protein
PFL: Possible flag leaf
PL: Palea
PSL: Pseudo spikelet
R: Root
RH: Rhizome
SAM: Shoot apical meristem
SDP: Short-day plant
SE: Standard error
TFL1: TERMINAL FLOWER1
TOC1: TIMING OF CAB EXPRESSION1
YLF: Young leaf from flowering culm
YLN: Young leaf from non-flowering culm
ZTL: ZEITLUPE
