# Supplementary material for: Identification, characterization and gene expression analyses of important flowering genes related to photoperiodic pathway in bamboo
Source: BMC Genomics. 2018 Mar 10;19:190. doi: 10.1186/s12864-018-4571-7 (PMC5845326; doi:10.1186/s12864-018-4571-7)
Supplement: Supplementary file 1 — Table S1. A summary of the oligonucleotides used in this study for different purposes. (DOC 90 kb) [file 12864_2018_4571_MOESM1_ESM.doc]

Additional file 1: Table S1. A summary of the oligonucleotides used in this study for different purposes.

| **Target gene** | **Oligo name** | **Sequence (5'-3')** | **Purpose** |
| --- | --- | --- | --- |
| *FT* | F1-4_F | ATGGTCGGCGGGGACAGG | Gene amplification |
|  | F1-4_R | TCACCAGGGGTACATCCTTCTTCC |  |
|  | FDG25_F | GATCCDCTGGTGGTKGGBAGG |  |
|  | FDG500_R | TGGCAGTTGAAGTARACRGYGGC |  |
|  | 5'FTlk3-4_R | CCGTTGCGCATGCACAACATCAT |  |
|  | 3'FTlk3-4_F | TCGCCTCTCACTCTTAATTTATCTCTAG |  |
| *FT1,2* | FT_LK_1-2_F | TGGACCCAGATGCTCCAAGT | Expression analyses by real time RT-qPCR |
|  | FT_LK_1-2_R | CACATCACCTCTTGCCCAATG |  |
| *FT3,4* | FT_Lk_3-4_F | CTCAAGCCGTCCATGGTGGT |  |
|  | FT_Lk_3-4_R | GGCTTGGAGCATCTGGGTCT |  |
| *COA* | CDG477_F | TRYGAHGCRVAGGTGCAC | Gene amplification |
|  | CDG1912_R | TRGCGAAVCGDCCCTTGAYC |  |
|  | BT_CO_1300_5'F | ATGAATTATAATTTCARCAGCHMCGT |  |
|  | BT_CO_1300_5'R | ACACTCGCTCCTTTCCTTCTCTG |  |
|  | BT_CO_1300_3'F | TATGATTCGCATATTTCAGTGACCATTT |  |
|  | BT_CO_1300_3'R | TCAGAACCATGGAACGGTRCYRTA |  |
| *COA* | BT_COA_LK1_Q_F | CTGCTGCTCAACAAAGATTCT | Expression analyses by real time RT-qPCR |
|  | BT_COA_LK1_Q_R | CTCTTGGTTGTTGTCGATGTG |  |
| *COB* | CO_B_DG_F | TCGAGCCGGAGCTCCSYGGCC | Gene amplification |
|  | CO_B_DG _R | TGCCGGGCACGACGARCTCGTG |  |
|  | Bt_COB_lk_1_3'_F | CCGTTCGGCGACGCACTG |  |
|  | Bt_COB_lk_1_3'_R | CTAGAACGTCGGCACGAC |  |
| *COB* | Bt_COB_lk_1_GSP2 | GGGCAGGAGGTGCGACTACGACGA | Expression analyses by real time RT-qPCR |
|  | CO_B_Q _R1 | TGCCGGGCACGACGAACTCGTG |  |
| *TOC1* | T8G130_F | ATGGTGGGCACCAGCGAG | Gene amplification |
|  | TDG116_R | CTACTCTGGDGAAGAAACCATCTCTACCTC |  |
| *TOC1* | TOC1_QRT_F | AGGAACAAGGAACTGCGCCACATC | Expression analyses by real time RT-qPCR |
|  | TOC1_QRT_R | GTCTTCGCCGCCACACATG |  |
| *ZTL* | ZDG293_F | TGCAATGYAGAGGVCCGTTTG | Gene amplification |
|  | ZDG293_R | TTCRGTTAGCATCCACTCTTCTCC |  |
| *ZTL* | ZTL_QRT_F | TGTCACCCAGGGATATAGCATC | Expression analyses by real time RT-qPCR |
|  | ZTL_QRT_R | TGGTCAACTCTCTTGCTAGTCG |  |
| *LHY* | LDG212_F | AGTTGGARAAGGAAGCTATCAAYAATG | Gene amplification |
|  | L3G510_R | TCAGGTGGACGCTTCGCTCTC |  |
| *LHY* | LDG212_F | AGTTGGAGAAGGAAGCTATCAACAATG | Expression analyses by real time RT-qPCR |
|  | LHY5'R | AGATATGGACTGTTAGGTTTTCTT |  |
| *GI* | GDG1_F | ATGTCAGCTTCAAATGRGAAGTGG | Gene amplification |
|  | GI_QRT_R | TCTAAGGAGTTCAACAAGCTG |  |
| *GI* | GI_3'_F | CTACTCCAAGTGCTACGCAGAGGTTGCT | Expression analyses by real time RT-qPCR |
|  | GI_QRT_R | TCTAAGGAGTTCAACAAGCTG |  |
| *eIF4α* | eIF-4_F | ATGCTCTCCCGTGGTTTCAAG | Reference gene for real time RT-qPCR data normalization |
|  | eIF-4_R | CAAGGGTAAGCTCATCTCTCTTCAC |  |
