# Supplementary figures and images for: Identification, characterization and gene expression analyses of important flowering genes related to photoperiodic pathway in bamboo
Source: BMC Genomics. 2018 Mar 10;19:190. doi: 10.1186/s12864-018-4571-7 (PMC5845326; doi:10.1186/s12864-018-4571-7)

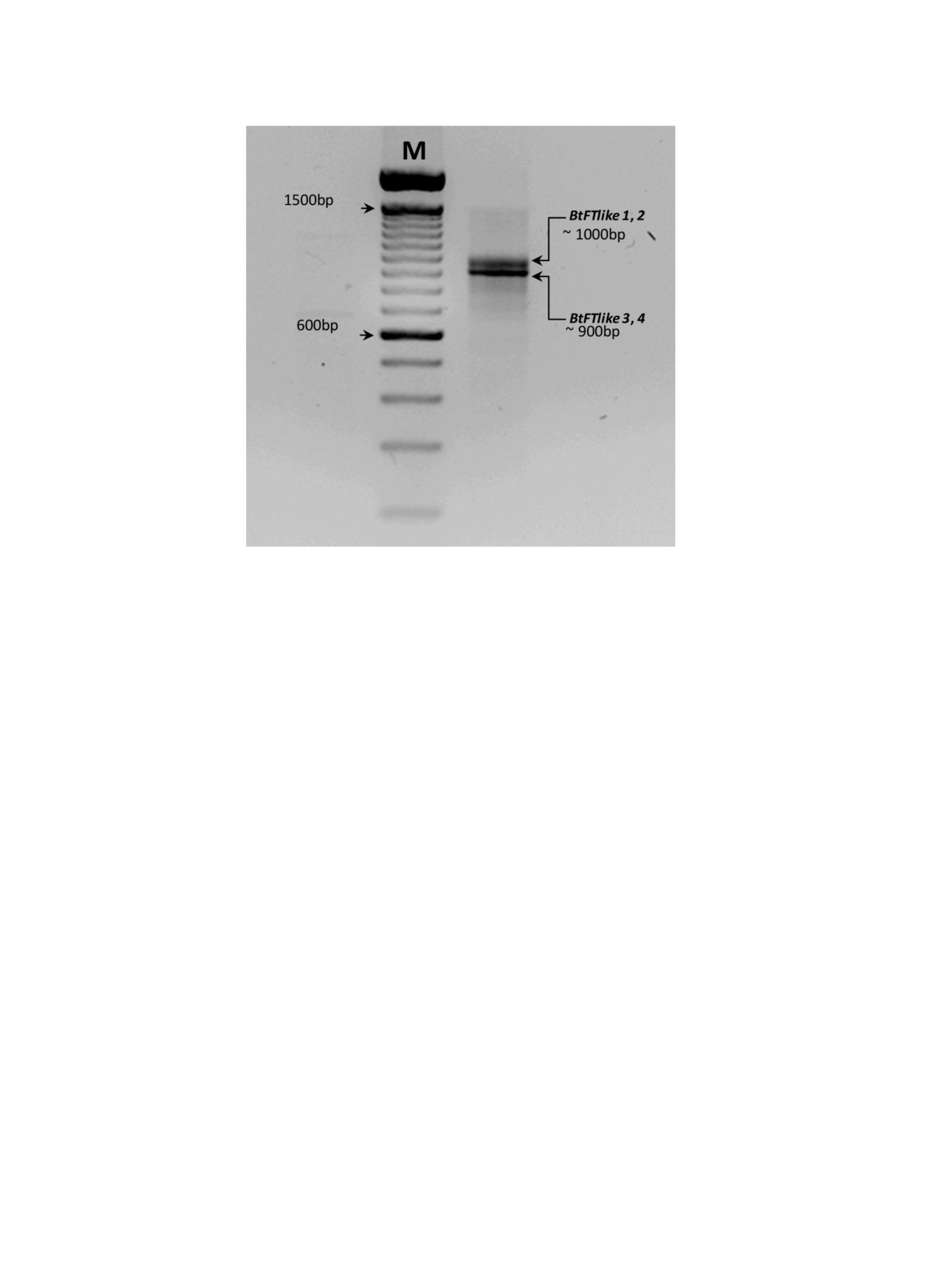

Supplement: Supplementary file 2 — Figure S1. Two PCR amplified bands using degenerate primers specific for FLOWERING LOCUS T (FT). (TIFF 472 kb) [file 12864_2018_4571_MOESM2_ESM.tif]
